# The influence of muscle pennation angle and cross-sectional area on contact forces in the ankle joint

**DOI:** 10.1177/0309324716669250

**Published:** 2016-09-22

**Authors:** Ran S Sopher, Andrew A Amis, D Ceri Davies, Jonathan RT Jeffers

**Affiliations:** 1 Department of Mechanical Engineering, Imperial College London, London, UK; 2 Musculoskeletal Surgery Group, Department of Surgery and Cancer, Imperial College London, London, UK; 3 Human Anatomy Unit, Department of Surgery and Cancer, Imperial College London, London, UK

**Keywords:** Ankle, muscle architecture, surface/deep pennation angle, physiological cross-sectional area, joint reaction forces

## Abstract

Data about a muscle’s fibre pennation angle and physiological cross-sectional area are used in musculoskeletal modelling to estimate muscle forces, which are used to calculate joint contact forces. For the leg, muscle architecture data are derived from studies that measured pennation angle at the muscle surface, but not deep within it. Musculoskeletal models developed to estimate joint contact loads have usually been based on the mean values of pennation angle and physiological cross-sectional area.

Therefore, the first aim of this study was to investigate differences between superficial and deep pennation angles within each muscle acting over the ankle and predict how differences may influence muscle forces calculated in musculoskeletal modelling. The second aim was to investigate how inter-subject variability in physiological cross-sectional area and pennation angle affects calculated ankle contact forces.

Eight cadaveric legs were dissected to excise the muscles acting over the ankle. The mean surface and deep pennation angles, fibre length and physiological cross-sectional area were measured. Cluster analysis was applied to group the muscles according to their architectural characteristics. A previously validated OpenSim model was used to estimate ankle muscle forces and contact loads using architecture data from all eight limbs.

The mean surface pennation angle for soleus was significantly greater (54%) than the mean deep pennation angle. Cluster analysis revealed three groups of muscles with similar architecture and function: deep plantarflexors and peroneals, superficial plantarflexors and dorsiflexors. Peak ankle contact force was predicted to occur before toe-off, with magnitude greater than five times bodyweight. Inter-specimen variability in contact force was smallest at peak force.

These findings will help improve the development of experimental and computational musculoskeletal models by providing data to estimate force based on both surface and deep pennation angles. Inter-subject variability in muscle architecture affected ankle muscle and contact loads only slightly. The link between muscle architecture and function contributes to the understanding of the relationship between muscle structure and function.

## Introduction

A muscle’s architecture (particularly its physiological cross-sectional area (PCSA), fibre pennation angle (PA) and optimal fibre length) is an established predictor of its force generation and excursion,^1^ in both musculotendon-actuator-^2,3^ and electromyography (EMG)-activation-data based^4^ models. Some models of musculoskeletal function (both experimental^5–10^ and computational^2,11–13^) apply the aforementioned models to calculate the distribution of load between different muscles, demonstrating that reliable data about muscle architecture are important in biomechanical research and clinical practice.

Human muscle architecture has been investigated using ultrasound^14–21^ and magnetic resonance imaging (MRI),^16,22,23^ but conventional machines are unable to measure individual muscle fibres. Given the limitations of current-generation medical imaging, the gold standard for obtaining muscle architecture data remains dissection of cadavers.^1^ Few studies have used cadaveric material to measure the architecture of the dorsiflexor, plantarflexor and peroneal muscles acting across the ankle joint;^24–28^ the two most cited studies^24,28^ investigated only two and three specimens. More recently, Ward et al.^27^ published a study of 21 lower limbs, which for the first time, provided data about the variation in muscle architecture. However, similar to previous studies,^24,28^ only surface measurements of muscle PAs were performed, despite the fact that there is evidence suggesting that there may be a difference between the PAs at the surface and the interior of a muscle,^29^ particularly for bi- or multi-pennate muscles with large PCSAs. Musculoskeletal models^12,25^ use a number of elements to simulate the forces exerted by muscles, particularly larger muscles, implying that it can be advantageous to use different architectural parameters, including PA, for each of these elements. This emphasises the potential benefit of obtaining PA data for both a muscle’s surface and its interior.

As implied above, inter-subject variability in muscle architecture is a source of uncertainty in biomechanical models involving application of simulated muscle forces. Such variability has been addressed within the framework of parametric sensitivity analyses, and its application is currently gaining popularity for use in musculoskeletal modelling.^30^ In view of the fact that differences in muscle architecture could affect estimates of force exerted by the muscle along its line(s) of action, which would, in turn, impact upon the outcomes of musculoskeletal modelling employing data of muscle architecture, the first aim of this study was to investigate potential differences between superficial and deep PAs within the muscles acting across the human ankle. The second aim was to investigate how inter-subject variability in muscle architecture affects ankle muscle and joint reaction forces (also referred to as talocrural contact loads) estimated through musculoskeletal modelling.

## Methods

### Measurements of muscle architecture

Eight formalin-embalmed cadaveric lower limbs from eight adult males (four right and four left; mean ± standard error of the mean (SEM) age of donors: 80 ± 5 years; height: 173 ± 1 cm), with no known anatomical abnormalities, were investigated in this study. All cadavers were donated and the study was performed at the Human Anatomy Unit, Charing Cross Campus, Imperial College London, in compliance with the provisions of the UK Human Tissue Act (2004).

The lower limbs were dissected to excise all muscles acting across the ankle: 1) the superficial plantarflexors: gastrocnemius, soleus and plantaris; 2) the deep plantarflexors: flexor hallucis longus (FHL), flexor digitorum longus (FDL) and tibialis posterior (TP); 3) the dorsiflexors: extensor hallucis longus (EHL), extensor digitorum longus (EDL) and tibialis anterior (TA) and 4) the peroneal muscles: peroneus longus (PL) and peroneus brevis (PB). For the purposes of the current study, peroneus tertius (PT) was included as a part of EDL, because of its small size and the fact that during dissections the muscle belly was not accurately separable from that of EDL.^31^

Individual muscle bellies were dissected from their tendons at the musculotendinous junction. Fat, nerves, blood vessels and fascia were removed from each muscle and care was taken to avoid cutting or stretching its fibres. Muscle body volumes were then measured using water displacement (±1 mL accuracy for the smallest muscles, to ±5 mL for the largest).

Muscle length was defined as the distance from the most proximal point of the muscle to the centre of its musculotendinous junction.^27,28^ It was measured from an overhead digital image of the muscle together with a millimetre scale, using an image-analysis script coded in MATLAB^®^ (version R2013a, MathWorks Inc., Natick, MA, USA).

**Figure 1.**
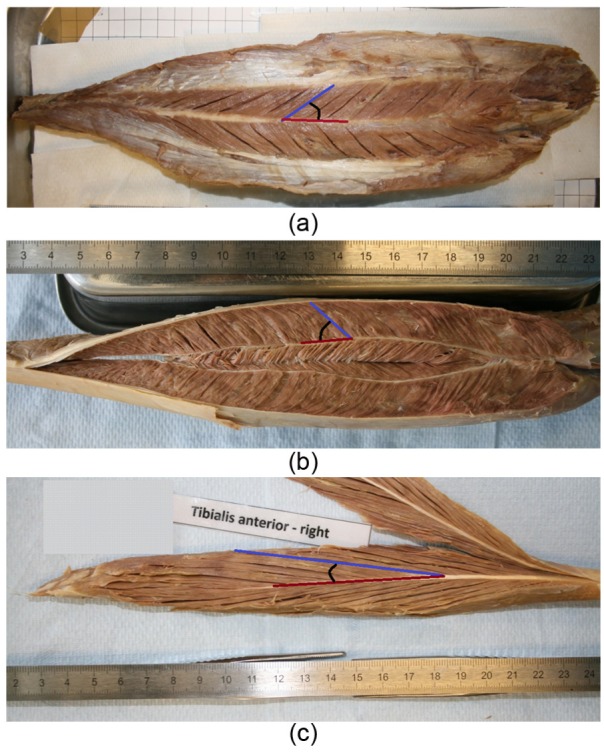
Photographs of example muscles ((a and b) soleus and (c) tibialis anterior). Fibre pennation angles (PAs) were measured on the muscle surfaces (a) before they were dissected to allow a clear visualisation of the fibres deep within the muscle (b and c). The images were used to estimate PA as the angle (black arc) between the muscle fibre (blue line) and tendon/aponeurosis (dark red line). The tibialis anterior (c) is an example for a muscle with long fibres and small PA, whereas the soleus (a) is the muscle with the shortest fibres and the largest PA. The difference in architecture between the soleus surface (a) and deep within it (b) is also demonstrated.

To measure surface PA, bundles of superficial muscle fibres were gently separated from each other using fine forceps and a scalpel, to allow better visualisation of their orientation. Digital images of the muscle surface were then taken from directly above using a photo-copy stand (Figure 1). The PAs of superficial muscle fibres with an approximately even distribution along the muscle length were subsequently measured using an image-analysis script coded in MATLAB and recorded to the nearest degree. The PA was defined as the angle between the line connecting the tendinous insertion and end points of the fibre, with the tangent to the tendon or aponeurosis at the fibre attachment point (Figure 1(a)).^16,32^

To measure PAs deep within a muscle, it was cut lengthwise along its tendon axis into two or three pieces in such a way that the muscle fibres were visualised in the plane of the cut.^33^ Groups of fibres distributed approximately evenly within the muscle were gently separated from each other and digital images were taken (Figure 1(b) and (c)) to allow measurement of PAs of deep muscle fibres as described above (measurements of the deep PAs were not possible in the plantaris, because it was too small and thin to be cut as described). The mean combined PA for each individual muscle was then calculated as the average of its mean surface and deep PAs.

To determine fibre length, muscle fascicles were dissected from each muscle with an approximately even distribution along its length.^24,26,28^ The fascicles were immersed in 20% w/w nitric acid for 48–96 h to digest connective tissue and allow separation of approximately 0.1-mm-thick bundles (the thinnest bundles we were able to isolate intact) from the two tendinous intersections or aponeurotic fascia bands between which they extended, with the aid of a dissecting microscope.^24^ Fibre lengths were estimated by directly measuring bundle lengths using a millimetre scale, while holding the bundles straight without stretching. The mean normalised fibre length was calculated as the mean fibre length divided by the length of the whole muscle.^24,28^

The PCSA of each muscle was calculated by dividing the muscle volume by its mean fibre length as reported previously.^16,24,26,34^ Normalised PCSA was calculated by dividing the muscle PCSA by the sum of PCSAs of all ankle-crossing muscles of that leg; normalised PCSAs may be used to estimate absolute PCSAs based on other anthropometric characteristics and also allow consistent comparison between subjects.^24,35^ Reduced PCSA, indicative of the force that a muscle can apply along its line of action, was calculated as the product of its PCSA and cosine of the mean combined PA.^23,27,28,36^ The anatomical cross-sectional area (ACSA) of each muscle was calculated by dividing its volume by its measured length.^36^

Data were tested for normality (Shapiro–Wilk). Two-way analysis of variance (ANOVA) with Bonferroni correction for post hoc analysis was used to explore the interaction between different muscles, the location of PA measurement (surface or deep) and the value of mean PA. One-way ANOVA and Kruskal–Wallis one-way analysis of variance tests were used to investigate differences in the mean fibre length, mean surface PA, mean deep PA, mean combined PA, volume and PCSA between different muscles. Mann–Whitney tests were used to identify significant differences between groups of muscles. Significance was set at *P*<0.05. After visually identifying three clusters of data points in a three-dimensional (3D) scatter diagram of the volumes, the mean fibre lengths and mean combined PAs of all muscles examined, the *k*-means clustering method^37^ was used to group data, with the number of clusters set to 3. All data-analysis techniques were applied using SPSS^®^ Statistics (Version 21; IBM Corp., NY, USA) and MATLAB. The plantaris muscle was excluded from the analysis due to the difficulty of acquiring deep PA measurements, as well as its minor role in controlling ankle movement.

To determine the test–retest reliability of the mean surface and deep PA measurement method, the procedure described above was repeated five times over five consecutive days using a soleus muscle from a randomly selected cadaver. The measurements were then analysed using the Fisher original intraclass correlation coefficient (ICC). Analysis revealed high reliability, with a mean PA test-retest difference of less than 3° and ICCs of .91 and .80 for the surface PA and deep PA, respectively.

### Musculoskeletal modelling

A previously validated musculoskeletal model implemented in OpenSim^11^ (Gait2392^38^) was adapted to estimate ankle muscle forces and contact loads applying the muscle architecture data acquired as described above. The musculoskeletal model, which is based on the pioneering work by Delp et al.,^2^ has 23 degrees of freedom and 92 musculotendon actuators (with adjustable architectural parameters) to simulate the kinematics and dynamics of the two lower limbs, pelvis and torso. Focusing on the foot and ankle, separate segments are assigned to the following rigid bodies onto which the muscles are attached: (1) tibia and fibula, (2) talus, (3) calcaneus, navicular, cuboid, cuneiforms and metatarsals and (4) phalanges. The talocrural and subtalar joints are both modelled as frictionless revolute joints allowing only flexion and version, respectively. Muscles modelled include those dissected (apart from the plantaris): gastrocnemius (two elements), soleus, FHL, FDL, TP, EHL, EDL, PT, TA, PB and PL. Muscle paths are adjusted using via points and wrapping surfaces where such are necessary to simulate the physiological scenario and prevent muscle lines of action from passing through bone as the joint moves. The scaled version of the model used herein represents a subject that is approximately 180 cm tall and has a mass of 72.6 kg; the musculotendon architectural parameters (including PCSA, PA and optimal fibre length) are derived from two of the aforementioned studies.^24,28^ Muscle isometric strength is considered proportional to the PCSA,^2,39^ normally assuming specific tension (muscle tensile stress, which is the force exerted by the muscle per unit of PCSA) of 61 N/cm^2^ as described previously.^2,38–40^ This value falls within the range of 35–137 N/cm^2^ that has previously been reported^41^ to only marginally influence muscle and joint reaction forces predicted through musculoskeletal modelling.

The simulation was set based on the reported kinematics and ground reaction forces occurring during self-selected speed level walking of an adult healthy male;^38,42^ the data are downloadable as part of a tutorial package available in the SimTk Project website.^43^ In view of the fact that the forces generated by the muscles acting over the ankle largely determine the joint reaction force at the talocrural joint, these forces were calculated in the OpenSim environment applying the static optimisation approach described previously^11,44,45^ (and implemented in similar studies estimating contact forces occurring in the hip, for example^13,46^) and utilising the Thelen^47^ muscle model, while assuming that the problem was static at each frame. Model outputs were calculated utilising the model’s nominal muscle architecture values,^24,28^ as well as the muscle PA and PCSA data from each of the eight lower limbs investigated in this study to create subject-specific model variants; other parameters (including optimal fibre length, which was not measured in this study; see below) were not altered and kept at their nominal values. The forces generated by EDL and PT were assumed to act through the EDL for the reasons described above; accordingly, the actuator simulating the EDL was assigned PCSA representing the sum of the PCSAs of the two muscles, while that simulating the PT was disabled. Contact loads occurring in the talocrural joint were then calculated. During this procedure, kinematic data, together with all external forces and estimated muscle forces, are used by the software to calculate the resultant load at the joint through dynamic analysis.^43,48^

## Results

### Measurements of muscle architecture

The sample means and SEMs of muscle volumes, lengths, mean fibre lengths, mean normalised fibre lengths, mean fibre surface PAs, mean fibre deep PAs, mean fibre combined PAs, ACSAs, PCSAs, reduced PCSAs and normalised PCSAs are listed in Table 1.

**Table 1.** Mean outcome measures (muscle volumes, lengths, mean fibre lengths, mean normalised fibre lengths, mean fibre surface PAs, mean fibre deep PAs, mean fibre combined PAs, ACSAs, PCSAs, reduced PCSAs and normalised PCSAs) for all muscles investigated. The peroneus tertius was considered a part of extensor digitorum longus. Values represent sample means ± standard errors of the mean. ACSA: anatomical cross-sectional area; N/A: not available; PA: pennation angle; PCSA: physiological cross-sectional area.

Muscle	Volume (mL = cm^3^)	Length (cm)	Mean fibre length (cm)	Mean normalised fibre length (cm)	Mean fibre surface PA (°)	Mean fibre deep PA (°)	*p*-Value for the difference between surface and deep PA^a^	Mean fibre combined PA (°)	ACSA (cm^2^)	PCSA (cm^2^)	Reduced PCSA (cm^2^)	Normalised PCSA (%)
Superficial plantarflexors												
Gastrocnemius	145 ± 18	25.2 ± 0.8	4.0 ± 0.2	0.16 ± 0.007	20 ± 4	18 ± 2	1	18 ± 2	5.8 ± 0.7	36 ± 5	34 ± 4	17 ± 1.2
Soleus	224 ± 25	30.7 ± 0.9	2.3 ± 0.1	0.07 ± 0.005	40 ± 3	26 ± 3	0.006*	32 ± 3	7.5 ± 1.0	98 ± 9	82 ± 8	47 ± 1.1
Plantaris	4 ± 1	10.6 ± 1.1	4.2 ± 0.3	0.41 ± 0.023	9 ± 1	N/A	N/A	N/A	0.3 ± 0.05	0.9 ± 0.1	0.9 ± 0.1	0.4 ± 0.1
Deep plantarflexors												
Flexor hallucis longus	36 ± 3	22.8 ± 0.9	3.4 ± 0.1	0.15 ± 0.009	20 ± 2	18 ± 2	1	19 ± 2	1.6 ± 0.2	11 ± 1	10 ± 1	5 ± 0.5
Flexor digitorum longus	18 ± 2	26.0 ± 0.8	3.5 ± 0.1	0.13 ± 0.002	15 ± 1	15 ± 2	1	16 ± 2	0.7 ± 0.1	5 ± 1	5 ± 1	2 ± 0.2
Tibialis posterior	56 ± 5	28.9 ± 0.6	2.8 ± 0.1	0.10 ± 0.003	17 ± 1	16 ± 1	1	17 ± 1	1.9 ± 0.2	20 ± 1	19 ± 1	10 ± 0.9
Dorsiflexors												
Extensor hallucis longus	18 ± 2	26.3 ± 0.5	7.5 ± 0.4	0.28 ± 0.013	10 ± 1	8 ± 1	1	10 ± 1	0.7 ± 0.1	3 ± 0.3	2 ± 0.3	1 ± 0.1
Extensor digitorum longus	42 ± 5	35.0 ± 0.6	6.9 ± 0.4	0.20 ± 0.011	12 ± 1	8 ± 1	0.755	11 ± 1	1.2 ± 0.1	6 ± 1	6 ± 1	3 ± 0.3
Tibialis anterior	65 ± 5	28.2 ± 1.2	6.6 ± 0.2	0.24 ± 0.010	13 ± 1	10 ± 1	0.430	11 ± 1	2.3 ± 0.2	10 ± 1	10 ± 1	5 ± 0.2
Peroneals												
Peroneus brevis	22 ± 3	23.2 ± 1.0	3.4 ± 0.2	0.15 ± 0.011	18 ± 1	13 ± 1	0.387	16 ± 1	0.9 ± 0.1	7 ± 1	7 ± 1	3 ± 0.4
Peroneus longus	44 ± 4	26.5 ± 0.7	3.7 ± 0.3	0.14 ± 0.008	19 ± 3	12 ± 1	0.104	16 ± 2	1.7 ± 0.1	13 ± 2	12 ± 2	6 ± 0.5

a After applying Bonferroni correction (multiplying the *P*-value by the number of comparisons, that is, 10).

There was a significant interaction between muscle type, location of PA (surface or deep) and mean PA (*P* = 0.009). Post hoc analysis revealed that for soleus, the mean (±SEM) surface PA was significantly greater than the mean deep PA (40° ± 3° compared with 26° ± 3°; *P* = 0.006 after Bonferroni correction; Table 1). There was no significant difference between the mean surface PA and mean deep PA for any of the other muscles investigated (*P* > 0.05 for all muscles).

There was a strong linear correlation (0.7 ≤ R^2^ ≤ 1) between PCSA and ACSA for gastrocnemius, FDL, TP, EHL, PB and PL, and a moderate linear correlation (0.4 ≤ R^2^ < 0.7) for soleus, FHL, EDL and TA (Table 2). A positive linear relationship was observed between the mean deep and surface PAs of soleus (R^2^ = 0.60)

$$\begin{array}{c} PA_{d}(\circ )=0.679\times PA_{s}(\circ )\\ 21{}^{\circ}<PA_{d}<43{}^{\circ},34{}^{\circ}<PA_{s}<54{}^{\circ}\;(R^{2}=0.60) \end{array}$$

where *PA_s_* and *PA_d_* are the surface and deep PAs, respectively.

**Table 2.** Linear curve fitting coefficients describing the relationship between PCSA and ACSA for each muscle (PCSA = *a* × ACSA, where *a* is the coefficient). R^2^ values, along with ranges of ACSA and PCSA for which the relationship was derived, are also indicated. ACSA: anatomical cross-sectional area; PCSA: physiological cross-sectional area.

Muscle	Linear curve fitting coefficient (*a*)	ACSA range (cm^2^)	PCSA range (cm^2^)	R^2^
Gastrocnemius	6.3	3.1–9.1	22.0–65.5	0.85
Soleus	12.5	4.6–13.6	75.1–152.2	0.44
Flexor hallucis longus	6.6	1.2–2.6	7.3–15.7	0.48
Flexor digitorum longus	7.5	0.5–1.1	3.2–8.4	0.98
Tibialis posterior	10.3	1.4–2.9	13.1–27.4	0.83
Extensor hallucis longus	3.6	0.5–1.0	1.4–4.1	0.83
Extensor digitorum longus	5.0	0.8–1.8	4.1–8.8	0.53
Tibialis anterior	4.2	1.8–3.2	6.6–13.6	0.63
Peroneus brevis	7.6	0.6–1.5	3.9–14.0	0.86
Peroneus longus	8.0	1.2–2.4	9.0–22.7	0.70

**Figure 2.**
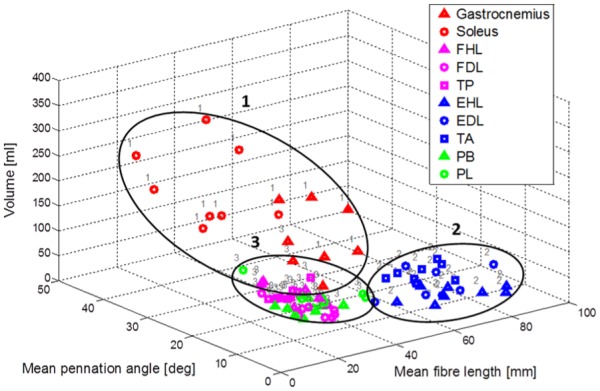
A scatter diagram showing volume, mean fibre length and mean PA (combined) from all 80 muscles examined in the study, with data points visually classified into three clusters (black ovals) identified as (1) superficial plantarflexors (gastrocnemius, soleus), (2) dorsiflexors (EDL – extensor digitorum longus; EHL – extensor hallucis longus; TA – tibialis anterior) and (3) deep plantarflexors (FDL – flexor digitorum longus; FHL – flexor hallucis longus; TP – tibialis posterior) and peroneals (PB – peroneus brevis; PL – peroneus longus). The grey labels indicate the outcome of a single iteration of the *k*-means clustering algorithm, which in this iteration was in agreement with the visual observations in 76 of 80 data points.

**Figure 3.**
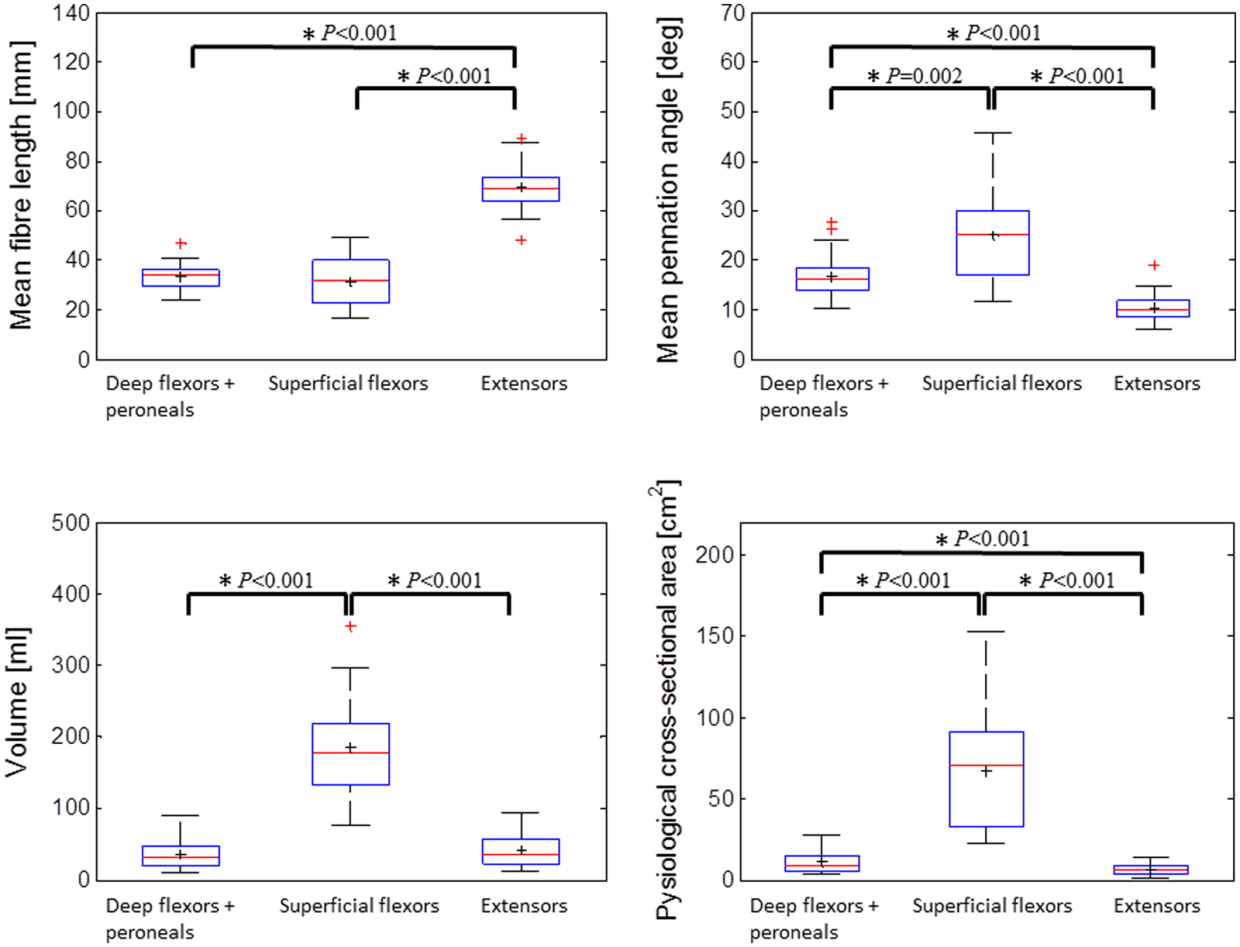
Box-and-whisker plots comparing medians (red bands), means (black ‘+’ signs) and ranges (interquartile ranges, blue boxes; most extreme data points not considered outliers, black whiskers; outliers, red ‘+’ signs) of data (mean fibre length, mean combined PA, volume and PCSA) collected from all 80 muscle specimens examined in the study and classified into three clusters (deep flexors/plantarflexors + peroneals, superficial flexors/plantarflexors, and extensors/dorsiflexors, as visualised in Figure 2). Asterisks indicate significant differences between clusters according to the Mann–Whitney test (*P* < 0.05).

Three clusters of data points were identified by analysis of a scatter diagram of the volumes, mean fibre lengths and mean combined PAs of all 80 muscles examined, utilising the *k*-means method (Figure 2). These clusters were (1) superficial plantarflexors (gastrocnemius, soleus), (2) dorsiflexors (EDL, EHL, TA) and (3) deep plantarflexors (FDL, FHL, TP) and peroneals (PB, PL). Only 4/80 points were assigned by the algorithm to a cluster different than that of their compartment. Significant differences in combined PA, fibre length, volume and PCSA were found between the three muscle groups (Mann–Whitney test *P* < 0.05; Figure 3).

### Musculoskeletal modelling

**Figure 4.**
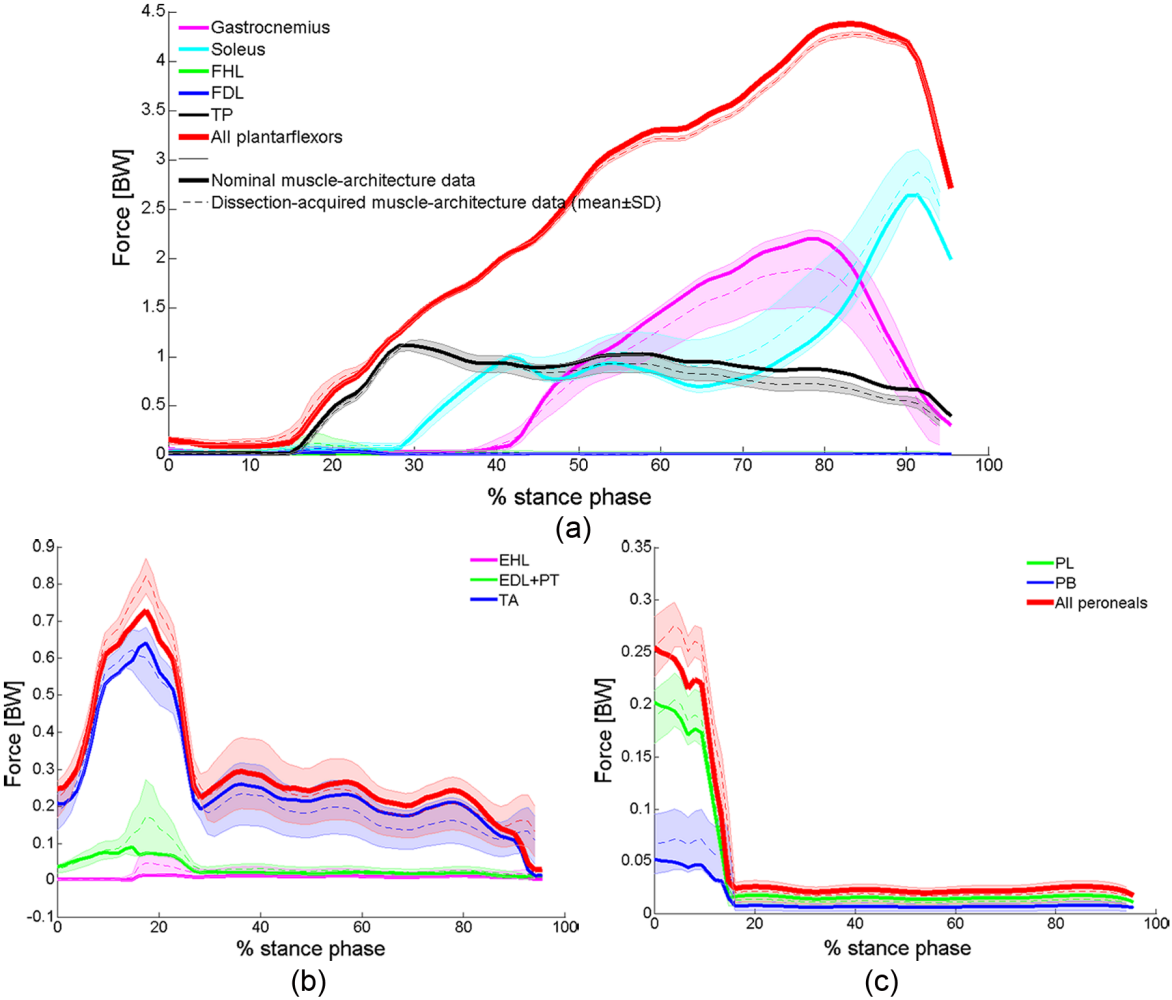
Plots showing estimates of muscle forces (normalised according to bodyweight, BW) exerted during a single cycle of the stance phase of gait. Data were derived from the OpenSim simulation, implementing the nominal PA and PCSA values used in the OpenSim Gait2392 model (thick lines) and those obtained from dissections of eight cadaveric specimens (mean forces across all specimens: dashed lines; ±1 standard deviation: shaded area). Forces produced by the ankle (a) plantarflexors, (b) dorsiflexors and (c) peroneals are plotted separately. EDL: extensor digitorum longus; EHL: extensor hallucis longus; FDL: flexor digitorum longus; FHL: flexor hallucis longus; PB: peroneus brevis; PL: peroneus longus; PT: peroneus tertius; TA: tibialis anterior; TP: tibialis posterior.

Plots of estimates of muscle forces acting during the stance phase of gait (normalised to bodyweight, BW), obtained with the OpenSim static optimisation algorithm using the PA and PCSA values acquired from the eight limbs dissected in the current study, together with those obtained using the nominal values of the OpenSim model, are given in Figure 4. Gastrocnemius and soleus were the two muscles generating the largest forces, with the peak values occurring before toe-off (~75% stance phase for gastrocnemius, ~92% stance phase for soleus), each exceeding two times BW (Figure 4(a)). The total force generated by the dorsiflexors was estimated by the model to peak just when mid-stance starts (~17% stance phase) at 0.7–0.9 BW (depending on the architectural properties applied; Figure 4(b)), with the TA accounting for at least 75% of that force. The forces exerted by the peroneals were even smaller, with peak total force equivalent to or smaller than 0.25 BW at heel strike (0% stance phase; Figure 4(c)). Differences between muscle forces calculated assuming different architectural properties were generally minor, as demonstrated in Figure 4. The smallest difference (approximately 2% at peak force) was calculated for the total force generated by the plantarflexors, which formed the muscle group predicted to exert the largest forces (Figure 4(a)).

**Figure 5.**
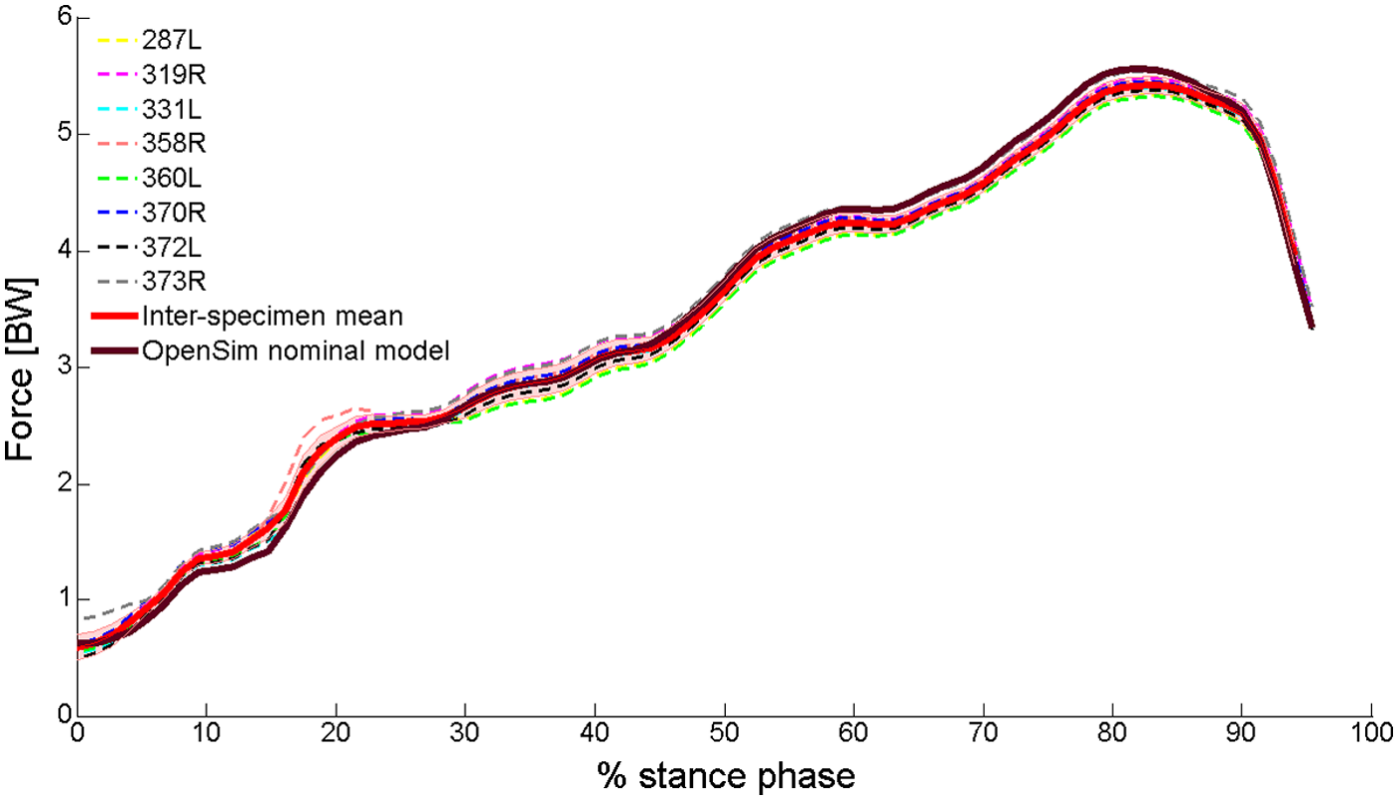
Plots showing estimates of the total contact force (normalised according to BW) acting in the talocrural joint during a single cycle of the stance phase of gait. Data were derived from the OpenSim simulation, implementing the nominal PA and PCSA values used in the OpenSim Gait2392 model (thick wine-red line) and those obtained from dissections of eight cadaveric specimens (dashed lines in various colours; the thick red line indicates the mean across all specimens, while the shaded area represents ±1 standard deviation; legend labels indicate the specimen numbers).

Plots of the model-predicted total reaction force acting at the talocrural joint during the stance phase (normalised according to BW) are shown in Figure 5. Differences between contact forces calculated assuming different architectural properties were minor. For all specimens, peak force occurred before toe-off (75%–83% stance phase), with a magnitude larger than five times BW (at 75% stance phase, for example, inter-specimen mean contact force: 5.0, standard deviation: 0.1, range: 4.9–5.1 BW). During the earlier stance phase, however, when predicted contact forces were smaller, differences between outputs obtained for different architectural properties were larger (e.g. an approximately 15% difference between the largest and smallest estimates at 20% stance phase).

## Discussion

Knowledge of a muscle’s architecture is important for predicting the forces that it generates, which is crucial in experimental and computational biomechanical modelling, including musculoskeletal models.^27,39^ This study is the first to compare surface with deep muscle fibre PAs, revealing a significant difference between those of soleus, with the mean surface PA being significantly greater (by 54%) than the mean deep PA (Table 1). This would result in a 15% underestimation of the force exerted by soleus (which is one of the strongest muscles acting over the ankle) when calculated using surface rather than deep PA measurements and assuming that the muscle force is proportional to the cosine of the PA.^4^

In this study, the PCSAs of the muscles acting over the ankle joint were 3.7- to 12.3-fold greater than their ACSAs (Table 2), in agreement with previous findings.^23,36^ The values derived for individual muscles can be useful for estimating a muscle’s PCSA from its ACSA, which can be measured easily in vivo using ultrasound or MRI.^23,35,49^ In order to test this claim, we used the mean muscle volume and muscle length data reported in one of these studies^35^ (only gastrocnemius, soleus, FHL, FDL, TP and TA, for which data were available), together with the ACSA-to-PCSA values that we derived, to estimate the muscle PCSAs; these were compared to PCSA estimates obtained through dividing the muscle-volume-to-muscle-length ratio from Handsfield et al.^35^ by the optimal fibre-length-to-muscle-length ratio from Ward et al.^27^ (as implemented in Handsfield et al.^35^). Estimates made according to the former approach were similar to those obtained through the latter one, though 10%–40% larger (Supplementary Table 2), which is within the range of the inter-specimen variability in muscle volume (Table 1).

There are two principal findings to the musculoskeletal modeling in the current study; the first is that a reaction force greater than five times BW was calculated for the talocrural joint between the heel rise and toe-off phases of the gait cycle (Figure 5); the second is that inter-limb variation in muscle PCSA and mean fibre PA (manifested in both variations between the cadaveric specimens used in this study and variations between these and the nominal muscle architecture values of the OpenSim model adapted in this study) only marginally affected ankle contact forces, including those acting between the heel rise and toe-off. The muscle forces that generated this joint reaction force were affected by inter-limb variation to a greater extent than the joint reaction forces. Yet, the total force generated by the plantarflexors, the muscle group exerting the largest forces acting over the ankle, demonstrated inter-subject variation of only 2% at peak force. This finding is supported by the model suggested by Zajac,^3^ demonstrating that the effects of fibre PA on musculotendon forces are significant only in especially large (i.e. larger than 23°) PAs (where all combined PAs measured in this study, excluding that of soleus, were smaller than 20°). This can explain the minor effect that the variation in muscle architecture had on peak contact force. To our knowledge, this is the first time that contact forces occurring in the ankle have been calculated using a contemporary musculoskeletal modelling platform (such as OpenSim, AnyBody^®^ or LifeModeler^®^) while exploring the sensitivity of the model outcomes to muscle architectural characteristics of PCSA and fibre PA.

The muscle architecture data (Table 1) are generally in good agreement with those reported in similar studies available in the literature (detailed comparison with previous cadaveric studies is available in Supplementary Table 1).^24–28^ Specifically, trends in the current data are consistent with previous studies, showing that soleus has the largest combined PA while plantaris, followed by the dorsiflexors, has the smallest. The dorsiflexors have the longest fibres and soleus, followed by gastrocnemius, have the largest volume and PCSA. However, for all muscles investigated in this study, the mean fibre lengths and mean normalised fibre lengths were shorter than those reported previously. This difference may be due to fibre lengths being ‘normalised’ according to sarcomere lengths (to give optimal fibre lengths) in two of the previous studies.^26,27^ Additionally, in this study, combined PAs were consistently found to be larger than those reported in the literature, which may be linked to differences in measurement techniques (using image analysis rather than a palpator). It should further be noted that the muscle architectural characteristics directly related to the muscle size (volume, ACSA and PCSA) reported here are typically 1.5–2 times smaller than in previous studies employing MRI to explore the leg musculature in young, healthy and/or physically active subjects.^23,35,49,50^ This discrepancy – attributable to pre-death muscle atrophy (as a result of sarcopenia, inactivity and pathology) in the cadavers dissected in this study^35^– was compensated in our musculoskeletal model with using a value of muscle specific tension (61 N/cm^2^) higher than reported in the literature, as suggested previously.^39,40^

The muscle and joint reaction forces calculated in this study are largely similar to those reported in the literature,^51–54^ including studies implementing musculoskeletal modelling in the OpenSim environment.^41,55^ In particular, the current estimates of both the total and vertical components of the reaction forces acting in the talocrural joint through the stance phase of gait were consistent with those in the literature, both in trend and magnitude.^41,55,51–53^ Of special interest is that for nearly all studies, peak force occurred before toe-off, with magnitude above five times BW. The exception for this is the study by Procter and Paul,^53^ which estimated the total talocrural contact force to peak at approximately 3.5 BW. This lower finding could be due to their model disregarding the inertial contributions of the bone components to the joint reaction forces, and assuming that antagonist muscle groups do not act simultaneously, which may not be the physiological reality. The study by Hardt^54^ similarly predicted the total ankle contact force to peak at 3.5 BW; however, this force was calculated as the sum of muscle forces predicted utilising the optimisation-based musculoskeletal model developed by the researcher, and inertial and gravitational forces reported in another study (and not calculated by Hardt). Also, one of the assumptions of the optimisation algorithm was that only few of the muscles modelled were active at any time point, which possibly resulted in an underestimation of the calculated contact forces. A recently published OpenSim model of the lower limb by Valente et al.^41^ predicted the largest muscles acting over the ankle to generate forces similar to those calculated in this model (gastrocnemius and soleus generating peak forces larger than 2 BW before toe-off, and TA generating force equivalent to approximately 0.6 BW when mid-stance starts). This model also predicted peak ankle contact force to be only slightly higher than in this study (approaching 6 BW). Perturbations in model inputs aimed at investigating the sensitivity of muscle and joint-reaction forces to uncertainties in muscle specific tension and musculotendon geometry affected the calculated muscle and joint reaction forces only to a moderate extent, which is in a further agreement with the findings of this model. A very recent subject-specific OpenSim model by Prinold et al.^55^ predicted peak ankle reaction forces to equal 4.5 or 6 BW (depending on the subject) and to be more sensitive to perturbations in muscle attachments, but these were calculated for three adolescents suffering from juvenile idiopathic arthritis rather than healthy adults.

The OpenSim simulation conducted in this study estimated contact forces occurring in other joints, including the hip joint. The latter were in a quantitatively and qualitatively good agreement with hip contact forces calculated in a previous experimentally validated study.^13,46^ The current finding that muscle force estimations are considerably more sensitive to variations in muscle PCSAs than joint contact forces also concurs with the results of a previous study employing musculoskeletal modelling of the hip joint.^56^

A limitation of this study may be the use of formalin-fixed specimens, because embalming may have affected muscle architecture. However, Cutts^57^ reported that embalming caused only ~2% shrinkage of excised leg muscles, indicating that the effect of fixation on architecture is marginal. Additionally, muscle PCSA, fibre length and PA in vivo are affected by joint position and muscle activity,^3,14,16,17,19^ giving an inherent limitation to the use of cadaveric material. The specimens investigated in this study were from elderly donors, in whom muscle architectural characteristics (particularly those related to the muscle size, including volume, ACSA and PCSA) could be different to those in a younger population.^20,34,58–60^ Also, all cadavers investigated in this study were from male donors and these exhibit differences (manifested particularly in the above characteristics) compared with females.^35,49,60–62^ However, differences in muscle mass between males and females have been shown to be significantly smaller in the lower than in the upper limbs.^61^ Additionally, sarcomere lengths were not measured in this study despite the fact that they are needed to calculate the optimal fibre length – in which the muscle is assumed to produce maximum force.^1,27^ A muscle’s PCSA was accordingly calculated as the fracture of the muscle volume and its mean fibre length^16,24,26,34^– rather than optimal fibre length.^25,27^ However, sarcomere length data available in the literature are of high accuracy and demonstrate that they are fairly consistent for the musculature acting over the ankle (2.12–3.24 µm^27^ or 1.89–3.16 µm^26^). Yet, the modelling presented here neglected the muscles’ force–length relationship and its effect on the muscle isometric strength. Similarly, the PA used as input for the OpenSim model should be that measured at optimal fibre length, and therefore it was assumed that the cadaveric PA values applied to the model in the current study were measured at optimal fibre length. These assumptions, however, are unlikely to have had an impact on the main conclusion of this study, according to which inter-limb variation in muscle PCSA and PA affects ankle-crossing muscle forces to a small extent and talocrural contact forces only marginally; also, a previous study^45^ found that static-optimisation-predicted hip, knee and ankle joint contact forces were affected by force–length–velocity constraints to a marginal extent, which were also implemented in a recent study.^41^ Finally, the architectural properties of the tendons (e.g. slack and taut lengths and cross-sectional areas, or length of the entire musculotendon unit) and muscle moment arms were also not measured despite the fact that they can contribute to the accuracy of musculoskeletal modelling.^2,39,63^ However, high-fidelity datasets of tendon architecture and muscle moment arms are already available in the literature.^64–67^ The architectural properties of the medial and lateral heads of gastrocnemius are not reported here separately, because the border between them is difficult to distinguish in the muscle belly, and attempts to investigate the heads separately in two cadavers did not reveal any difference in mean fascicle length or fibre PA. Therefore, the same PA and PCSA values were assigned to both elements modelling this muscle in the musculoskeletal model.

The main limitations of the musculoskeletal model employed in this study are associated with the use of gait data from a single subject (although we did implement subject-specific muscle architectural properties). Also, when adjusting the model according to experimentally measured, subject-specific PCSA and PA, the model and gait data were not re-scaled. However, since one of the aims of this study was to explore the effect of the muscle architectural properties on the reaction forces in the talocrural joint, it was expedient to adjust these properties according to the experimental data we acquired, while keeping all other model inputs at their nominal values. Also, in view of the fact that the data used as inputs for the current OpenSim simulation are characteristic of normal level walking of adult healthy males,^38,42^ the joint reaction load estimations produced by the simulation are likely to be representative of the ‘normal’ physiological condition. Another potential limitation of the model is demonstrated in the considerable differences found between dorsiflexor and peroneal muscle activation profiles as predicted by this model and clinical EMG data available in the literature.^4^ Despite the fact that qualitative agreement between EMG data and muscle activation profiles has been reported in several studies,^3,13,68,69^ it has also been demonstrated that surface EMG is not necessarily a reliable predictor of the forces exerted by smaller and/or deeper muscles.^4,68^ Accordingly, differences similar to those we report here have been previously found in musculoskeletal models of the knee,^48,70^ for example. These differences can be attributed to EMG being unable to predict passive musculotendon forces (those applied by taut tendons), which are accounted for in the current musculoskeletal model. More importantly, it has also been demonstrated that the musculoskeletal- modelling-predicted activations of the leg musculature (including some muscles acting across the ankle) are affected by the objective function used in the static optimisation algorithm and can therefore substantially deviate from EMG profiles.^13,70^ A further important limitation of the current contact load estimation is the lack of experimental data to verify the outcomes obtained through computational modelling (where such data collected using instrumented joint replacements are available for the hip, knee and shoulder joints^71,72^ and have been used to validate model-predicted joint contact forces occurring in the hip^13^ and knee^48,70^).

## Conclusion

Computational and experimental modelling are crucial research approaches in musculoskeletal research, since ethical and practical considerations limit the collection of data in vivo. The muscle-architecture data reported here may be used to estimate muscle forces through musculoskeletal modelling, which can contribute to the understanding of the roles of the leg muscles and assist in surgical decision-making (e.g. tendon transfer) and ankle prosthesis design. Additionally, this study is the first to demonstrate a difference between surface and deep PAs and provide evidence to group the muscles acting over the ankle joint according to their architecture, thus identifying links with their functional roles. The current study is also one of the first to demonstrate that joint reaction forces calculated in a musculoskeletal model are relatively insensitive to subject-specific muscle PCSA and fibre PA; however, there was a considerable variation in the soleus muscle force calculated for surface compared with deep PA.

Future investigations of muscle architecture should be expanded to include data from female and younger subjects. Also, since the muscle architectural properties assessed in this study are affected by joint position and muscle activity, measurement of muscle architecture in vivo by applying advanced imaging techniques can provide data to improve future musculoskeletal models. Future work could also include a multi-subject study in which the musculoskeletal model described herein would utilise subject-specific gait data to more accurately predict contact forces occurring in the ankle.

## Supplementary Material

Supplementary material

## Supplementary Material

Supplementary material
