# Supplementary material for: The influence of muscle pennation angle and cross-sectional area on contact forces in the ankle joint
Source: J Strain Anal Eng Des. 2016 Sep 22;52(1):12–23. doi: 10.1177/0309324716669250 (PMC5952297; doi:10.1177/0309324716669250)
Supplement: Supplementary material [file Supplementary_Table_1.pdf]

Supplementary Table 1: The main outcomes of this study (mean fibre length, mean normalised fibre length, mean PA, muscle volume, PCSA and normalised PCSA) compared with these of previous cadaveric studies (Friederich and Brand, 1990; Klein Horsman et al., 2007; Spoor et al., 1991; Ward et al., 2009; Wickiewicz et al., 1983). Differences (percentage-wise) are also provided. EDL – extensor digitorum longus, EHL – extensor hallucis longus, FDL – flexor digitorum longus, FHL – flexor hallucis longus, Gast – gastrocnemius, PB – peroneus brevis, PL – peroneus longus, TA – tibialis anterior, TP – tibialis posterior.

		**Gast.**	**Soleus**	**Plantaris**[i]	**FHL**	**FDL**	**TP**	**EHL**	**EDL**	**TA**	**PB**	**PL**
**Literature** (28 ≤ n ≤ 30)	(Optimal) fibre length [mm]	53	38	44	48	41	34	76	72	70	43	47
	Normalised fibre length	0.22	0.10	0.48	0.19	0.15	0.11	0.31	0.24	0.26	0.18	0.17
	Pennation angle [deg]	12	30	4	17	13	14	9	10	9	11	13
	Volume [cm^3]	181	269	6	39	19	56	20	39	79	25	55
	PCSA [cm^2]	35	71	1.4	9	5	18	3	6	12	6	12
	Normalised PCSA	20%	40%	1%	5%	3%	10%	2%	3%	7%	4%	7%
**This study** (n = 8)	Fibre length [mm]	40	23	42	34	35	28	75	69	66	34	37
	Normalised fibre length	0.16	0.07	0.41	0.15	0.13	0.10	0.29	0.20	0.23	0.15	0.14
	Pennation angle [deg]	18	32	9	19	16	17	10	11	11	16	16
	Volume [cm^3]	145	224	4	36	18	56	18	42	65	22	44
	PCSA [cm^2]	36	98	0.9	11	5	20	3	6	10	7	13
	Normalised PCSA	17%	47%	0.4%	5%	2%	10%	1%	3%	5%	3%	6%
**% Difference**	Fibre length	-24%	-40%	-4%	-29%	-15%	-17%	-1%	-5%	-6%	-20%	-22%
	Normalised fibre length	-27%	-26%	-16%	-20%	-12%	-14%	-9%	-19%	-10%	-20%	-20%
	Pennation angle[ii]	+53%	+7%	115%	+11%	+26%	+18%	+10%	+5%	+23%	+48%	+24%
	Volume	-20%	-17%	-35%	-9%	-6%	0%	-9%	+7%	-18%	-12%	-20%
	PCSA	+3%	+38%	-36%	+28%	+4%	+14%	+5%	+5%	-15%	+11%	+8%
	Normalised PCSA	-15%	+16%	-44%	+2%	-27%	0%	-39%	-8%	-25%	-16%	-13%

[i] Not all studies report data of the plantaris (only Klein Horsman et al., 2007; Spoor et al., 1991; Wickiewicz et al., 1983 do), and thus n=7.
[ii] Mean PA combining surface and deep PA.

1. **Friederich, J.A., Brand, R.A., 1990. Muscle fiber architecture in the human lower limb. Journal of Biomechanics 23 (1), 91-95.**
2. **Klein Horsman, M.D., Koopman, H.F., van der Helm, F.C., Prose, L.P., Veeger, H.E., 2007. Morphological muscle and joint parameters for musculoskeletal modelling of the lower extremity. Clinical Biomechanics 22 (2), 239-247.**
3. **Spoor, C.W., van Leeuwen, J.L., van der Meulen, W.J., Huson, A., 1991. Active force-length relationship of human lower-leg muscles estimated from morphological data: a comparison of geometric muscle models. European Journal of Morphology 29 (3), 137-160.**
4. **Ward, S.R., Eng, C.M., Smallwood, L.H., Lieber, R.L., 2009. Are current measurements of lower extremity muscle architecture accurate? Clinical Orthopaedics and Related Research 467 (4), 1074-1082.**
5. **Wickiewicz, T.L., Roy, R.R., Powell, P.L., Edgerton, V.R., 1983. Muscle architecture of the human lower limb. Clinical Orthopaedics and Related Research (179), 275-283.**
