# Supplementary material for: The influence of muscle pennation angle and cross-sectional area on contact forces in the ankle joint
Source: J Strain Anal Eng Des. 2016 Sep 22;52(1):12–23. doi: 10.1177/0309324716669250 (PMC5952297; doi:10.1177/0309324716669250)
Supplement: Supplementary material [file Supplementary_Table_2.pdf]

Table 2: Linear curve fitting coefficients describing the relationship between PCSA and ACSA for each muscle (PCSA=*a**ACSA, where *a* is the coefficient). R^2 values, along with ranges of ACSA and PCSA for which the relationship was derived, are also indicated.

**Muscle**	**Linear curve fitting coefficient (*a*)**	**ACSA range [cm^2]**	**PCSA range [cm^2]**	**R^2**
Gastrocnemius	6.3	3.1-9.1	22.0-65.5	0.85
Soleus	12.5	4.6-13.6	75.1-152.2	0.44
Flexor hallucis longus	6.6	1.2-2.6	7.3-15.7	0.48
Flexor digitorum longus	7.5	0.5-1.1	3.2-8.4	0.98
Tibialis posterior	10.3	1.4-2.9	13.1-27.4	0.83
Extensor hallucis longus	3.6	0.5-1.0	1.4-4.1	0.83
Extensor digitorum longus	5.0	0.8-1.8	4.1-8.8	0.53
Tibialis anterior	4.2	1.8-3.2	6.6-13.6	0.63
Peroneus brevis	7.6	0.6-1.5	3.9-14.0	0.86
Peroneus longus	8.0	1.2-2.4	9.0-22.7	0.70
